# CDKN1A/p21 in Breast Cancer: Part of the Problem, or Part of the Solution?

**DOI:** 10.3390/ijms242417488

**Published:** 2023-12-14

**Authors:** Evangelos Manousakis, Clàudia Martinez Miralles, Maria Guimerà Esquerda, Roni H. G. Wright

**Affiliations:** Basic Sciences Department, Faculty of Medicine and Health Sciences, Universitat Internacional de Catalunya, 08195 Barcelona, Spain

**Keywords:** p21, CDKN1A, cyclin-dependent kinase inhibitor 1A, breast cancer, cell cycle, cancer stem cells

## Abstract

Cyclin-dependent kinase inhibitor 1A (Cip1/Waf1/CDKN1A/p21) is a well-established protein, primarily recognised for its pivotal role in the cell cycle, where it induces cell cycle arrest by inhibiting the activity of cyclin-dependent kinases (CDKs). Over the years, extensive research has shed light on various additional mechanisms involving CDKN1A/p21, implicating it in processes such as apoptosis, DNA damage response (DDR), and the regulation of stem cell fate. Interestingly, p21 can function either as an oncogene or as a tumour suppressor in these contexts. Complicating matters further, the expression of CDKN1A/p21 is elevated in certain tumour types while downregulated in others. In this comprehensive review, we provide an overview of the multifaceted functions of CDKN1A/p21, present clinical data pertaining to cancer patients, and delve into potential strategies for targeting CDKN1A/p21 as a therapeutic approach to cancer. Manipulating CDKN1A/p21 shows great promise for therapy given its involvement in multiple cancer hallmarks, such as sustained cell proliferation, the renewal of cancer stem cells (CSCs), epithelial–mesenchymal transition (EMT), cell migration, and resistance to chemotherapy. Given the dual role of CDKN1A/p21 in these processes, a more in-depth understanding of its specific mechanisms of action and its regulatory network is imperative to establishing successful therapeutic interventions.

## 1. Introduction

### 1.1. Medical Need for Targeted Therapeutics and Biomarkers for the Management of Late-Stage Metastatic Breast Cancer

Breast cancer is the most prevalent cancer type, accounting for over 2 million cases in 2020 [1]. The accumulated knowledge in recent decades has resulted in advancements in treatment strategies, including radiotherapy, surgery, chemotherapy, and immunotherapy [2,3]. Nevertheless, the effectiveness of these treatment options significantly varies between late-stage (TNM stage IV) and early-stage (TNM stage I and II), with a 5-year survival rate around 30% for advanced stages, compared to 90–100% for tumours detected earlier [4,5]. Unfortunately, 20–30% of patients initially diagnosed at early stages eventually succumb to recurrent metastatic tumours, which exhibit heightened aggressiveness and drug resistance compared to primary tumours [6,7]. Therefore, there is a need for novel small molecules and innovative technologies/strategies aimed at specific factors driving the metastatic process.

In addition to the improvement of therapy options for metastasis, the identification of predictive biomarkers for patient stratification, and the early detection of metastatic spread would not only alleviate the burden on healthcare systems but also significantly enhance the quality of life for women suffering with metastatic breast cancer. In this review, we will focus on CDKN1A/p21, a protein central to key processes during metastatic spread, including cancer stem cell survival, epithelial–mesenchymal transition (EMT), and apoptosis, and discuss the described seemingly opposing roles [8] and potential uses as a predictive biomarker in metastatic breast cancer.

### 1.2. Cyclin-Dependent Kinase Inhibitor 1A

The cyclin-dependent kinase inhibitor 1A (CDKN1A, p21, CIP1, or WAF1) gene encodes for a potent cyclin-dependent kinase (CDK) inhibitor, the activity of which has been well documented regarding control of the mammalian cell cycle. Belonging to a larger family of cyclin-dependent kinase inhibitors, CIP/KIP proteins, CDKN1A/p21 can inhibit CDK activity both in vitro and in vivo via direct binding multiple CDKs and cyclin proteins [9,10]. Even though members of this family share a cyclin–CDK domain within the N-terminal, which aids in the inhibition of the cyclin–CDK complexes, they do not share additional sequence similarity, suggesting that they have unique functions and/or roles in cellular processes independent of the inhibition of CDKs and their role in cell cycle regulation.

**Figure 1:**
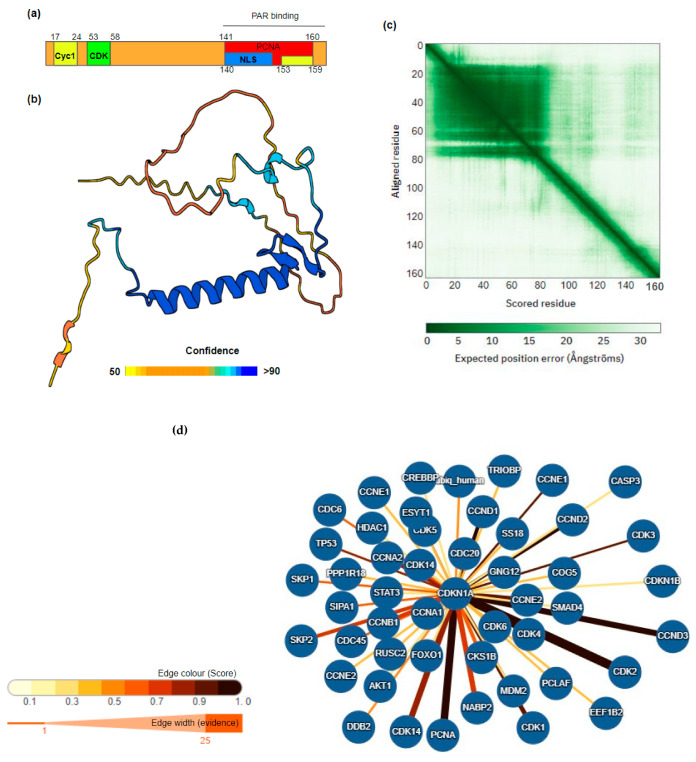
Structure and interactors of CDKN1A/p21. (**a**) Domain structure CDKN1A/p21; binding sites for cyclins (Cyc), CDK, PCNA, and poly-ADP-ribose (PAR) are indicated. (**b**) Alpha fold prediction of p21/CDKN1A 3D structure, confidence score scale bar is shown below, the amino and larger C-terminal disordered domains are clearly visible. (**c**) Predicted aligned error (PAE) for each residue [11]. (**d**) Annotated human protein interactors of CDKN1A/p21 using IntAct molecular interaction database. Score and evidence weights are shown by colour width of interaction edges [15] as shown in the legend. Full interaction data are detailed in Supplementary Table S1.

CDKN1A/p21 is a 164 amino acid protein, with a cyclin-dependent kinase inhibitor domain containing the cyclin- and CDK-binding motifs within the N-terminal and a nuclear localisation signal and binding sites for the replication protein, PCNA, and poly-ADP-ribose (PAR) within the C-terminal (Figure 1a–c). In addition, in terms of its 3D structure and organisation CDKN1A/p21 contains a disordered domain at the N-terminal and a longer C-terminal disordered domain (Figure 1b). Intrinsically disordered proteins (IDPs) and proteins containing large stretches of disordered regions play important roles in cell signalling, response to stimulus, and gene expression reprogramming as the disordered nature of this part of the polypeptide chain allows for the flexibility of binding, meaning that the protein can interact with multiple interaction partners depending upon the stimulus or pathway activated. The presence of these disordered regions in CDKN1A/p21 suggests that CDKN1A/p21 functions and interactions may be more complex than the canonical role in cell cycle regulation.

Although expressed in all mammalian tissues, p21 shows elevated expression signatures in the brain, connective tissue, and female reproductive tissues at the RNA and protein levels, respectively, clustering most significantly in smooth muscle tissue and extracellular matrix organisation [11]. At the cellular level, CDKN1A/p21 is located primarily within the nucleoplasm and nuclear bodies in normal cells, with the exception of monocytes [12,13], although cytoplasmic localisation has been observed in certain conditions and cancers, the significance of which will be later discussed.

Given the key role played by CDKN1A/p21 in the cell cycle, tight regulation of CDKN1A/p21 activity is essential. Regulation occurs via direct expression modulation but also via extensive post-translational modification. PTMs include phosphorylation, acetylation, methylation, and ubiquitination. These modifications result in a wide range of changes, including movement in terms of cellular localisation, increased protein stabilisation or degradation, and altering protein–protein interaction partners (Table 1). Indeed, the anti-cancer properties of berberine have been shown to be partly due to the increased nuclear localisation and stability of CDKN1A/p21 [14]. Human interacting proteins of CDKN1A include proteins involved in the cell cycle, DNA damage, and apoptosis (Figure 1d).

**Table 1:** Post-translational protein modifications of CDKN1A.

Post-Translational Modification	Site	Effector	Observed Effect	Reference
Phosphorylation	Thr-55	MELK (mouse)	Induce interaction CDK2/CDK4	[16]
	Thr-57	MAPK1, MAPK14 and MAPK8	Cytoplasmic localisation and degradation	[17,18]
	Thr-80	LKB1	Degradation	[19]
	Thr-145	AKT1, CHEK1, DAPK1, PIM1, PIM2, PKA	Impairs binding to PCNA Enhances protein stability Alters protein localisation	[20,21]
	Ser-31	Unknown	Unknown/identified using mass spectrometry	[22]
	Ser-98	ASK1	Unknown	[23]
	Ser-114	GSK3-beta	Enhances ubiquitination	[24]
	Ser-123	Unknown	Protein stabilisation	[25]
	Ser-130	CDK6, MAPK, MAPK14, MAPK8	Impairs stability	[26,27]
	Ser 137	Unknown		[28]
	Ser-153	DYRK1B, PKCA	Cytoplasmic localisation	[29]
	Ser-160	PKC	Modulates binding to PCNA	[30]
	Ser-146	AKT, CHEK1, DAPK1, LATS2, NUAK1, PKC, PRKCD, STK38	Impairs binding to PCNA Protein stabilisation	[31,32]
	Tyr-151	Unknown	Unknown	[33]
Ubiquitination	Lys-16 Lys-75 Lys-141 Lys-154 Lys-161 Lys-163	Breast cancer cell growth-induced	Protein degradation	[34]
Acetylation	Lys-141 Lys-154 Lys-161 Lys-163	HDAC1, TSA induced, cell growth and carcinogenesis inhibited	Protein stabilisation enhanced following acetylation	[35]
Methylation	Arg-156	PRMT6	Induction of cytoplasmic localisation	[36]

## 2. Role of CDKN1A/p21 in Cancer

### 2.1. Clinical Significance of CDKN1A/p21 Expression Levels

#### 2.1.1. CDKN1A/p21 in Cancer Progression

**Figure 2:**
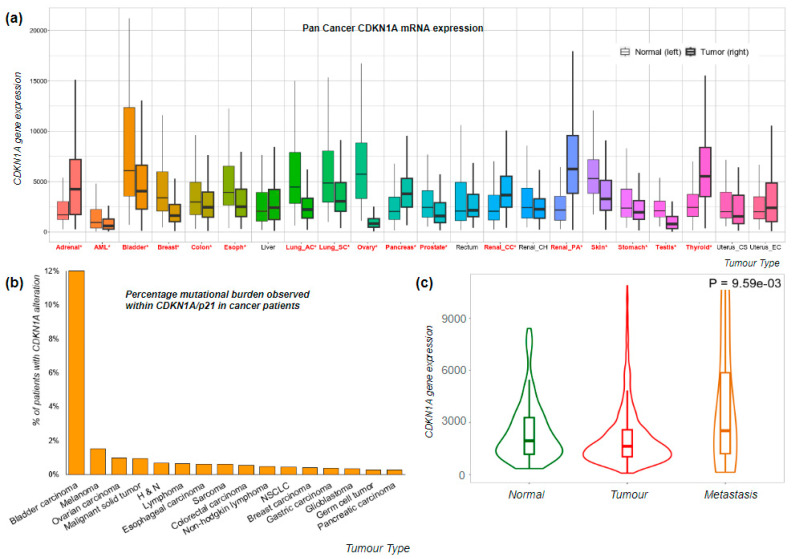
Expression of CDKN1A/p21 in pan and breast cancer datasets. (**a**) CDKN1A gene expression levels in pan cancer dataset [55], including 15,648 normal, 40,442 tumour, and 848 metastatic samples; significant differences according to Mann–Whitney U tests are marked in red with an asterisk. (**b**) CDKN1A/p21 alteration frequency detected including all alterations, mutations, amplification, and loss. (**c**) CDKN1A gene expression analysis, RNA-Seq; normal, *n* = 403, tumour, *n* = 1097, and metastatic, *n* = 7 (TCGA Project “Breast Invasive Carcinoma” analysed using TNMplot), Kruskal–Wallis test T vs. N 1.9 × 10^−51^, N vs. M 8.86 × 10^−3^.

The expression levels of p21 vary when comparing tumour versus normal tissue, showing differences both in magnitude and direction across various tumour types. Using TMPlot, a tool that integrates expression data from repositories like NCBI-GEO, The Cancer Genome Atlas (TCGA), Therapeutically Applicable Research to Generate Effective Treatments (TARGET), and Genotype-Tissue Expression (GTEX), we can observe distinct patterns. In certain cancer types, such as adrenal, pancreatic, and renal, there is a significant (*p* < 0.01) increase in CDKN1A/p21 expression in the tumour tissue compared to normal tissue, while in others, such as AML, colon, and lung, the opposite trend is observed (Figure 2a).

The role of CDKN1A/p21 in various cancer types has been extensively studied using cell lines, primary cultures, and patient samples (Table 2). Hypermethylation of the CDKN1A/p21 promoter [37,38,39,40,41] and the binding of other regulatory proteins such as SOX2 and LMNB2 [42,43] lead to increased cancer cell proliferation by silencing its expression. Conversely, stabilisation of CDKN1A/p21 at both the mRNA [44] and protein levels [45] promotes cell cycle arrest in cancer cells. However, CDKN1A/p21 has also been documented to promote cancer progression by stabilising cyclin D1–CDK4 complexes [46] and inhibiting apoptosis [47].

In mouse models, the deletion of CDKN1A/p21 does not affect carcinogenesis until around 16 months, where spontaneous tumours are observed [48,49]. Other phenotypes observed in CDKN1A/p21 knockout mice include morphological abnormalities during development, such as enlarged spleens, hearts, and smaller testes [50].

In terms of the mutational burden, CDKN1A mutations vary both in terms of the type of mutation and cancer. Overall, CDKN1A is altered in 0.96% of solid tumour malignancies [51] (Figure 2b). Among these, bladder carcinoma stands out with the highest mutation burden within CDKN1A/p21, affecting 11.99% of patients.

The cytoplasmic localisation of CDKN1A/p21 is predominantly associated with cancer, where it serves to promote tumorigenesis and inhibit apoptosis [52,53,54]. Notably, in breast cancer cell lines, the CDKN1A/p21 phosphorylation induced by the Akt pathway, especially in HER-2/neu-overexpressing cells, leads to cytoplasmic localisation. This event is central to cancer cell survival and resistance to apoptosis [52]. Moreover, the cytoplasmic translocation of CDKN1A/p21 in SUM159 breast cancer cells has been found to enhance chemoresistance [54].

In breast cancer patients, we observe a reduction in CDKN1A/p21 expression when compared to normal samples, although there is some variation among patients (Figure 2c). In addition, there is an increase in CDKN1A/p21 expression in metastatic patients compared to normal samples, although this observation is constrained by the limited number of metastatic cases sampled.

#### 2.1.2. Expression of CDKN1A/p21 Shows Predictive Promise in Breast Cancer

**Figure 3:**
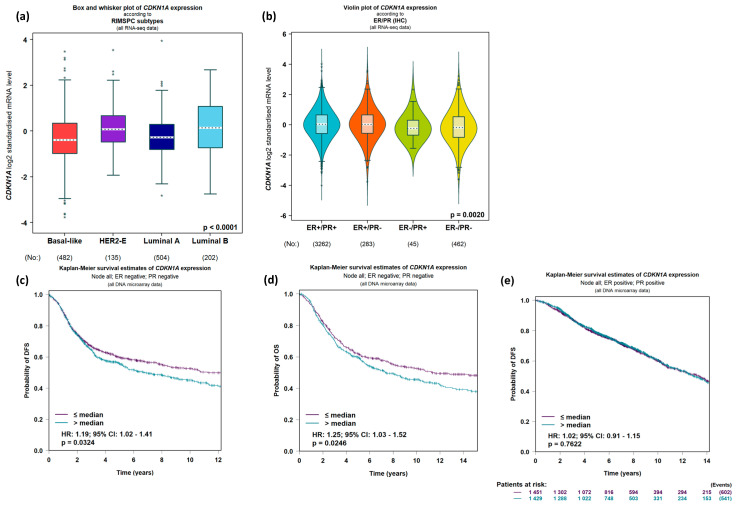
Expression levels and predictive power of CDKN1A/p21 in breast cancer patient datasets. Expression levels of CDKN1A log2 standardised mRNA grouping patients based on RIMSPC subtypes (**a**) or ER/PR expression levels detected using immunohistochemistry (IHC) (**b**); the number of patients in each dataset is indicated below each graph. (**c**) Disease-free survival (DFS) and overall survival (OS) analysis (**d**) of ER/PR-negative breast cancer patients stratifying patients based on high versus low expression levels of CDKN1A: significant *p* values based on CDKN1A of 0.0324 and 0.0246, respectively. (**e**) Disease-free survival (DFS) of ER/PR positive breast cancer patient dataset stratifying patients based on high versus low expression levels of CDKN1A. Data was analysed using GenExMiner 5.0.

Patient data analysis reveals that CDKN1A/p21 shows a notable preference for Luminal A type (Figure 3a) and HR (Hormone Receptor) subtypes following cancer patient stratification, which could hold significant implications for treatment options (Figure 3b). HR-negative patients, in particular, exhibit a markedly poorer overall prognosis in comparison to HR-positive breast cancer patient datasets. Within this subset of HR-negative patients, stratification based on p21/CDKN1A expression levels emerges as a potent predictor of not only diminished overall survival but also an elevated risk of recurrence and metastasis (Figure 3c,d). Conversely, such trends are not observed within the HR+ patient datasets (Figure 3e).

#### 2.1.3. Chemical Modulation of CDKN1A/p21 Expression and Action

One chemical modulator of CDKN1A/p21 is UC2288, which shares structural similarities with Sorafenib, a kinase inhibitor used in renal cancer therapy. Sorafenib, independent of its other targets, has been shown to reduce the protein levels of CDKN1A/p21 [56], which was also observed for UC2288 both at the mRNA and protein levels. By inhibiting cytosolic CDKN1A/p21, UC2288 impacts its anti-apoptotic function, resulting in decreased viability across various cancer cell lines [57]. Other small molecules, such as butyrolactone [58] and LLW10 [59], have also been shown to decrease CDKN1A/p21 protein levels, increasing degradation. However, their limited stability and lack of specificity make their use in clinical practice unlikely [60].

Histone deacetylase inhibitors (HDACis) represent promising agents in cancer therapy, inducing cell cycle arrest and apoptosis in diverse cell types [61,62]. Part of the mechanism of action of HDACis involves the upregulation of CDKN1A/p21 expression [63,64,65,66,67,68]. In one study, HDAC 2 knockdown using siRNA in HeLa cells resulted in increased CDKN1A/p21 expression at both the mRNA and protein levels, independently of p53 protein expression, leading to increased apoptosis [65]. Additionally, Trichostatin A (TSA), a well-known HDACi, induced the upregulation of apoptosis and growth arrest in the G0/G1 phase of human lymphatic endothelial cells (LEC), in a CDKN1A/p21-dependent and p53-independent manner [66]. In this case, the mRNA level of CDKN1A/p21 increased while the protein level remained constant; however, an accumulation of the protein within the nucleus instead of the cytoplasm was observed.

These observations highlight the dual role of CDKN1A/p21 in tumour growth. UC2288 and HDACi both enhance apoptosis, showing anti-tumour potential. TSA achieves this by increasing the expression levels of CDKN1A/p21, whereas UC2288 exerts its effects by inhibiting the CDKN1A/p21 function at the protein and cell localisation level. A more comprehensive understanding of their mechanisms of action may provide fresh insights into the role of CDKN1A/p21 in cancer progression.

### 2.2. Role of CDKN1A/p21 in Molecular Processes Important to Cancer Progression

#### 2.2.1. Cell Cycle

The relationship between cancer and cell cycle control is tightly interconnected, as any dysregulation in this process can lead to uncontrolled cell proliferation and tumorigenesis [69]. Targeting specific molecules involved in cell cycle regulation holds great promise in cancer therapy [70].

In this context, the role of the CDKN1A/p21 protein in cell cycle regulation has been well established for many years, yet it appears more complex than initially thought. Firstly, CDKN1A/p21 directly binds to cyclin-dependent kinases (CDKs), hindering the progression of the replication machinery [71,72]. This cell cycle arrest via CDK binding predominantly occurs in the G1 phase and partially in the G2 phase, where higher levels of CDKN1A/p21 correlate with the duration of these phases [73].

Secondly, in normal cells, CDKN1A/p21 forms a complex with CDK and proliferating cell nuclear antigen (PCNA). Even in the absence of CDKs, CDKN1A/p21 can inhibit PCNA-dependent activation of DNA replication [74]. CDKN1A/p21 directly binds to PCNA vis a PIP-Box (PCNA-interacting protein) region near the C-terminal, competing for the same site of PCNA with elements of DNA replication [75]. This interaction during the S-phase may block DNA replication after initiation, providing an alternative safety mechanism if the blockade in the G1 phase proves ineffective. However, PCNA is integral to the DNA damage response (DDR) [76], and thus complete inhibition of PCNA is not beneficial to the cell. Consequently, the interaction of CDKN1A/p21 with PCNA is more intricate. In fact, endogenous levels of CDKN1A/p21 are insufficient to block DNA replication in the S-phase [77].

CDKN1A/p21 can also indirectly influence the cell cycle via its involvement in gene regulation. The overexpression of CDKN1A/p21 in human cells leads to the downregulation of specific genes associated with mitosis and the upregulation of genes linked to senescence, including those encoding extracellular matrix (ECM) proteins [78]. This is achieved via a functional interplay of CDKN1A/p21 with transcription factors. For instance, c-Myc, a known transcription factor promoting mitosis, can directly bind to the carboxyl-terminal region of CDKN1A/p21, the same binding site shared with PCNA. This results in the release of CDKN1A/p21’s inhibitory effect on PCNA activity. Simultaneously, CDKN1A/p21, via binding to c-Myc, represses its transcriptional activity. The balance between these two activities can determine whether the cell progresses through the cell cycle [79].

The DREAM (dimerisation partner, RB-like, E2F, and multi-vulval class B) complex is a protein complex acting as a transcriptional repressor for a significant group of cell cycle genes, via a p53-dependent pathway. In the absence of CDKN1A/p21, the cyclin/CDK complexes hinder the formation of the complete DREAM complex, thereby preventing cell cycle repression. In the presence of CDKN1A/p21, cyclin/CDK complexes are inhibited, permitting the formation of a complete DREAM complex [80].

The retinoblastoma protein (RB) is a tumour suppressor that regulates the cell cycle by forming complexes with the E2F family of transcription factors. CDKN1A/p21 prevents the phosphorylation of RB, resulting in complex repression [81]. Furthermore, p53 binds to the promoters of genes related to cell cycle regulation. Immunoprecipitation assays have shown that the CDKN1A/p21-p53 interaction is essential for p53 to bind to the promoters of PUMA (BBC3), MDM2, and GADD45A [82]. This presents another way in which CDKN1A/p21 indirectly influences the cell cycle via p53-dependent gene regulation.

Moreover, research has shown that CDKN1A/p21 can act as a transcription factor itself. Chromatin immunoprecipitation assays revealed that CDKN1A/p21 binds via its N-terminal region to the transcriptional start site of a set of cell cycle genes, repressing mRNA expression [83]. Additionally, CDKN1A/p21 can directly bind to the enhancer of the SOX2 gene, regulating its activity [84]. These multifaceted roles that CDKN1A/p21 play in cell cycle control within the nucleus make it an intriguing target for cancer therapy; however, the various mechanisms of its action and regulation require further global gene expression, chromatin binding, and pathway analysis to facilitate more sophisticated drug development approaches.

#### 2.2.2. CDKN1A/p21 Is a Key Player in the DNA Damage Response

The DNA damage response (DDR) is a crucial cellular process responsible for safeguarding genomic stability, and defects in this process are a hallmark of tumorigenesis [85]. Given its paramount significance to cell health, many proteins involved in the DDR are targets of drug discovery projects for the treatment of cancer, particularly via the exploitation of deficits in DDR within cancerous cells and inducing synthetic lethality [86].

CDKN1A/p21 has been proposed as a regulator of the cellular response to DNA damage. Firstly, by directly inhibiting cell cycle progression, it affects the cell’s ability to repair the DNA before replication resumes. DNA damage being triggered by external sources prompts an increase in CDKN1A/p21 expression, primarily via a p53-dependent pathway [73,87]. Secondly, CDKN1A/p21 also affects the repair process via binding to PCNA [88,89], which in turns affects the interaction of PCNA with other proteins involved in the DNA repair processes [90]. The role of CDKN1A/p21 in the DNA damage response is dual. In general, CDKN1A/p21 inhibits the response mechanisms by binding to PCNA and preventing binding to regulatory proteins important for the completion of repair, but on the contrary, the presence of CDKN1A/p21 seems to promote repair [91] or regulate the repair process, for example, in the case of BER [92].

**Figure 4:**
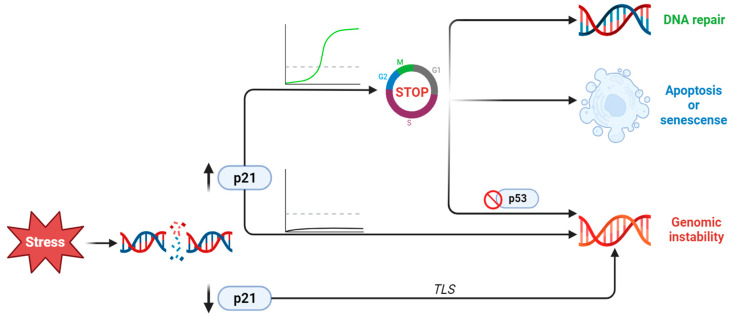
Summary of CDKN1A/p21 actions following DNA damage exposure. The protein levels of CDKN1A/p21 after damage may increase or decrease, leading to different outcomes. The decrease or increase under a certain threshold keeps the cell in the cell cycle without any repair of the damage and thus leads to genomic instability. Increases over the threshold may also lead to different outcomes. Initially, the cell arrests the cell cycle in order to repair the damage. If subsequently CDKN1A/p21 levels drop, repair continues. If the levels of CDKN1A/p21 remain high, then the cell becomes senescent, permanently exiting the cell cycle, or enters apoptosis.

Cell cycle arrest is critical during the DNA damage response (DDR) [93], therefore making CDKN1A/p21, a cell cycle inhibitor, pivotal in this process. A study employing time-lapse imaging and single-cell tracking demonstrated that following damage, CDKN1A/p21 levels must rise above a threshold before the cell enters the S phase, pausing the cell cycle [94]. An interesting exception is Translesion DNA Synthesis (TLS), where pol η is incorporated and binds to proliferating cell nuclear antigen (PCNA). In the absence of replication stress, p21 binds to PCNA, preventing it from interacting with pol η, inhibiting TLS [95]. Furthermore, after DNA damage caused by UV, methylmethane sulfonate, or hydroxyurea, CDKN1A/p21 levels decrease, and TLS takes place [96]. At the single-cell level, it has been observed that after the initial increase via the p53 pathway, CDKN1A/p21 is actively degraded during the S phase to prevent genomic instability, as high levels of CDKN1A/p21 inhibit DNA repair [97]. When CDKN1A/p21 levels remain high, the cell may enter a state of senescence or apoptosis. Notably, Panagiotis Galanos et al. demonstrated that during sustained expression of CDKN1A/p21 in the absence of p53, a subpopulation arises that re-enters the cell cycle, a phenomenon known as re-replication stress, which can lead to genomic instability [98]. In conclusion, the precise regulation of p21 levels is a crucial aspect of the DNA damage response (DDR) (Figure 4).

Members of the poly-ADP-ribose polymerase (PARP) family, such as PARP1-3, play a vital role in DDR, from the recognition of DNA lesions to chromatin remodelling and repair [99]. PARP1’s involvement in Base Excision Repair (BER) has been extensively studied [100]. In human fibroblasts, the loss of CDKN1A/p21 results in increased chromatin binding of PARP1, leading to an impaired DDR after exposure to DNA-damaging agents. CDKN1A/p21 directly binds to the N-terminal region of PARP1, which encompasses the DNA binding and automodification domains [101]. CDKN1A/p21 also positively regulates the binding of PARP1 to other BER intermediates, as demonstrated in vitro utilising photoreactive BER intermediates [102]. Additionally, PARP1 interacts with p53 [103,104]. p53 is PARylated within the C-terminal domain, which facilitates p53 recruitment to DNA damage sites, as observed in time-lapse experiments measuring the exogenous p53 in p53 null Saos2 cells [105,106]. p53 serves as an upstream regulator of CDKN1A/p21, and its regulation by PARP1 may also influence CDKN1A/p21 expression. Indeed, DDX11-AS1, a long non-coding RNA found to be upregulated in liver cancer patients, affects the interaction between p53 and PARP1, resulting in decreased CDKN1A/p21 expression at both the transcriptional and protein levels. This interaction was demonstrated using a human hepatocellular carcinoma (HCC) cell line [107]. Sustained expression of CDKN1A/p21 in human fibroblasts after DDR activates the MAPK14 and TGFb signalling pathways, which induces the production of reactive oxygen species (ROS). Additionally, ROS-induced DNA damage activates the DDR, creating a positive feedback loop which keeps the cells in growth arrest until the damage is repaired or the cell exits the cell cycle or undergoes apoptosis [108].

#### 2.2.3. CDKN1A/p21 Activation or Repression during EMT, Invasion, and Metastatic Transformation

Epithelial–mesenchymal transition (EMT) is a reversible process in which cells gradually transform from an epithelial-like phenotype to more a mesenchymal one. EMT is important during embryogenesis, tissue regeneration, and cancer. During EMT, a number of transcriptional factors, notably Snail1, TWIST (Twist Family BHLH Transcription Factor 1), and ZEB1/2, are activated, driving expression changes, which leads to alterations in the composition and structure of the cytoskeleton and cell adhesion molecules, a loss of cell junctions, and an increased invasive capability. During cancer progression, as the tumour grows, cells transition into mesenchymal phenotypes, which increases the metastatic ability, cancer stem cell (CSC) properties, and immuno- and drug-resistance [109,110,111,112].

It has been demonstrated in normal breast cell lines [113] and transgenic mice [114] that silencing of CDKN1A/p21 leads to the increased expression of EMT markers including TWIST and Snail1, decreased E-cadherin, and the induction of mesenchymal markers, suggesting that CDKN1A/p21 is a negative regulator of EMT, and may play an important role in the maintenance of the epithelial phenotype. CDKN1A/p21 downregulation is suggested to be necessary for Plasmacytoma Variant Translocation 1 (PVT1) lncRNA-induced EMT in triple-negative breast cancer cells [115] and in pancreatic cancer cells [116]. MiR-149-3p, a microRNA highly expressed in ovarian cancer patients, promotes EMT, in part by downregulating CDKN1A/p21 expression at both the RNA and protein levels [117]. In addition, loss of CDKN1A/p21 is associated with CMS4 colorectal cancer, an aggressive form of colorectal cancer characterised by the presence of mesenchymal-type cells [118].

The exact mechanism of CDKN1A/p21 regulation in EMT is not clear; however, there is emerging evidence to suggest an interplay between CDKN1A/p21 and important EMT transcriptional factors. CDKN1A/p21 forms a complex with ZEB1, inhibiting its action to repress miR-183-96-182, a miRNA that promotes EMT [119]. In another study, using lung carcinoma cells, it was shown that silencing of Snail1 leads to the upregulation of CDKN1A/p21 and cell cycle arrest. The cells also exhibited a more epithelial phenotype, decreased EMT marker expression, and reduced migratory capacity [120]. In contrast, during renal fibrosis, Snail1-induced EMT in animal models is accompanied by an upregulation of the p53–p21 axis and cell cycle arrest [121], suggesting that depending on the biological context, the role of CDKN1A/p21 may be different.

In triple-negative breast cancer, upregulation of the pro-inflammatory cytokine TNFα has been shown to be associated a more aggressive cancer type, via the activation of genes involved in invasion. An example is the metalloproteinase 9 (MMP9), which degrades ECM proteins in the local tumour environment via its proteolytic actions. Similar to MMP9, CDKN1A/p21 expression is upregulated in triple-negative breast cancer following TNFα exposure, which is not observed in normal cells. Importantly, MMP9 upregulation and subsequent increased invasiveness is directly dependent on the expression of CDKN1A/p21, indicating that CDKN1A/p21 may play a direct role in controlling the expression of key genes involved in EMT, invasion, and metastasis [122]. Angiogenesis is an important hallmark of cancer progression and consequently an interesting target for therapy [123]. However, the complexity and variety of mechanisms and pathways, even in the context of the same tumour, results in poor efficacy for anti-angiogenic treatments and acquired tumour resistance [124,125]. Concerning the role of CDKN1A/p21 in this process, studies suggest that CDKN1A/p21 in general promotes angiogenesis. The repression of CDKN1A/p21 in cancer cells decreases their angiogenic capacity in vitro by upregulating the angiogenic factor thioredoxin. Mechanistically, they demonstrated that CDKN1A/p21 binding to the promoter of thioredoxin-binding protein 2 (TBP2) (an inhibitor of thioredoxin) results in reduced TBP2 expression and thus increased thioredoxin [126]. More recently, it was shown that the long non-coding RNA MCM3A-AS1 carried in extracellular vesicles of cervical cancer cells promotes angiogenesis by upregulating CDKN1A/p21. The upregulation of CDKN1A/p21 is due to the binding of MCM3A-AS1 to microRNA-93, which when unbound, inhibits the expression of CDKN1A/p21 [127]. On the contrary, CDKN1A/p21 has been shown to also be able to repress the expression of the angiogenic factor VEGF in primary mouse embryonic fibroblasts (MEFs). Upon sustained hypoxia or DNA damage, CDKN1A/p21 is required for the p53-mediated downregulation of VEGF expression. In addition, CDKN1A/p21-null MEFs present increased angiogenic capacity compared with normal MEFs in xenotransplanted tumours [128]. Interestingly, using an in vivo model of retinal angiogenesis, it was shown that CDKN1A/p21 is spatially expressed in the tip endothelial cells (ECs) of the angiogenic vessels, accompanied by high VEGF stimuli, low Notch signalling, and high ERK signalling. This upregulation of CDKN1A/p21 results in a proportional increase in arrested ECs during highly mitogenic stimuli and consequently impaired angiogenesis. It appears that CDKN1A/p21 may serve as a defensive mechanism reducing EC proliferation and angiogenesis in the event that the ERK signalling pathway, which is known to induce proliferation, is activated [129]. Overall, the data suggest that throughout the process of metastatic progression, invasion, EMT, secondary site colonisation, and angiogenesis, CDKN1A/p21 may play an opposing role, depending on the cellular system, stimulus, and time of activation.

#### 2.2.4. Stem Cells

Cancer stem cells (CSCs) are a small population of cells residing initially within the primary tumour. CSCs are self-renewing (pluripotent) and are considered to be the initiators of tumorigenesis, metastasis, and recurrence [130]. CSCs are often resistant to traditional radio- and chemotherapy treatments [131]; given this and their key role in metastasis, especially for breast cancer, targeting breast cancer stem cells (BCSCs) is a promising target for novel drug discovery and biomarker projects [132,133].

Stem cell renewal and differentiation rely on the coordination between cell cycle progression and exit [134], and it is not surprising that CDKN1A/p21, as a regulator of those functions, plays an important role in the determination of stem cell fate, especially in cancer stem cells [135]. CDKN1A/p21 is also involved in the DNA damage response in adult stem cells, implying that it plays a role in the survival of cancer stem cells after chemotherapy and therefore resistance [136]. Activation of the p53–p21 pathway, for example, after oxidative stress, induces senescence in breast cancer stem cells [137]. Similarly, depletion of the stem cell marker Musashi-1 (MSI1) leads to the upregulation of CDKN1A/p21 and increased apoptosis in breast cancer cells [138]. Additionally, silencing of CDKN1A/p21 in mice and in vitro results in the upregulation of stem cell gene signatures [114].

On the contrary, irradiation of hematopoietic stem cells (HSCs) leads to an upregulation of CDKN1A/p21, independently of p53, protects the cells from apoptosis, and induces self-renewal [139]. In this case, it appears cells adopt a different mechanism in response to DNA damage, protecting them from apoptosis, dependent on the inhibition of p53. To further support this, mesenchymal stem cells (MSCs) deficient in CDKN1A/p21 become more tumorigenic if p53 is also depleted [140].

On the other hand, in basal conditions, CDKN1A/p21 may protect adult stem cells from proliferative exhaustion [141], not only by affecting entry into the cell cycle, which could lead to asymmetrical divisions and the loss of a self-renewal capacity [142], but also by directly downregulating pluripotency factors such as SOX2 in neural stem cells (NSCs) [84]. In the case of embryonic stem cells (ESCs), suppression of CDKN1A/p21 by TET1 is necessary for the maintenance of a proliferative state [143]. 

It is clear that depending on the cellular system and the developmental stage, CDKN1A/p21 may have contradicting roles in the determination of stem cell fate. In breast cancer, in which we often observe a downregulation of CDKN1A/p21, this may serve as a predictive marker for elevated stem-like properties in tumours, and thus increased probability of metastasis and recurrence. 

#### 2.2.5. Apoptosis and Senescence

Dysregulation of apoptotic pathways is known to be a hallmark of cancer, where tumour cells can evade apoptosis via various mechanisms [144,145]. In addition, dysregulation in apoptotic pathways can lead to tumours that do not respond to chemotherapy, resulting in higher rates of recurrence, due to the development of a resistant population [146]. Especially for breast cancer, chemoresistance is a growing concern [147,148]. 

While nuclear CDKN1A/p21 mainly regulates the cell cycle and DNA damage response, cytoplasmic CDKN1A/p21 can inhibit apoptosis by directly interacting with several apoptotic proteins [149,150]. It has been proposed that CDKN1A/p21 interacts with pro-caspase-3 [151] or apoptosis signal-regulating kinase1 (ASK1) [152], inhibiting their activity. CDKN1A/p21 protects HCT116 cells from IR-induced apoptosis, a function that is diminished in cells mutated within the CDK binding site of CDKN1A/p21 [153]. CDKs play an active role in apoptotic pathways [154,155]; thus, CDKN1A/p21 in the nucleus can inhibit the initiation of apoptotic pathways independently of the effects of the cytoplasmic protein. It has been shown that the p53/p21 complex can directly inhibit anti-apoptotic protein Bcl-2 and induce apoptosis in lung [156] and prostate cancer cells [157].

CDKN1A/p21 is a known partner of many transcription factors, some of which are mediators of apoptotic signalling, for example, c-Myc. CDKN1A/p21 can repress the activity of c-Myc, which in turn can repress the expression of CDKN1A/p21 by binding to its promoter [158]. C-Myc can activate apoptotic pathways via p53-dependent and p53-independent mechanisms [159] and therefore this balance may determine the decision between cell cycle arrest or apoptosis. 

All of the above suggests that the anti-apoptotic effect of CDKN1A/p21 is not as simple as previously thought, and it is not facilitated only by cytoplasmic CDKN1A/p21. Cytoplasmic CDKN1A/p21 may also induce apoptosis in certain cellular types. When the cell is faced with stress or DNA damage, CDKN1A/p21 is upregulated. The cell then either transiently arrests the cycle to allow the process of DNA repair to take place or permanently arrests and undergoes apoptosis. A lot of different mechanisms come into play considering arrest and repair or apoptosis, but the levels of CDKN1A/p21 and its cellular localisation seem to be two important factors. Senescence is a complex process with implications in development, ageing, and cancer [160,161,162,163]. Initially recognised for its anti-tumoural attributes due to the permanent cessation of the cell cycle, recent findings have suggested a potential oncogenic role: senescence may facilitate cellular migration and metastasis [164,165]. Notably, senescence induced by anti-cancer treatments emerges as a pivotal factor influencing recurrence and metastatic events [166,167,168], and key drivers of senescence are targets for cancer therapy drug development pipelines.

Considering senescence, the contradictory role of CDKN1A/p21 in cancer progression is important, as its upregulation induces a senescence-like phenotype in diverse cell lines [169,170,171]. The dual nature of senescence likely contributes to the perplexing role of CDKN1A/p21 in cancer biology. Pharmacological senescence was induced in non-small cell lung cancer cells using doxorubicin, and the expression level of CDKN1A/p21 was measured at the single-cell level. Intriguingly, cells exhibiting an early surge in CDKN1A/p21 levels (at 5.7 h post-treatment) entered a proliferative state, whereas those presenting a delayed increase (at 36 h) ended up in a senescent state. Notably, all senescent cells maintained elevated CDKN1A/p21 levels, whereas their proliferative counterparts reverted to lower levels [172]. In addition, it was demonstrated that cytoplasmic p21 may play a key role in CDK4/6 inhibitor-induced senescence in breast cancer cells [173]. These findings provoke intriguing ideas regarding the temporal dynamics of CDKN1A/p21 upregulation and its role in induced pharmacological senescence.

## 3. Discussion and Future Directions

In this review, we have discussed the implications of CDKN1A/p21 in cancer and present its different cell functions, which can both promote and suppress tumour growth. The dual role of CDKN1A/p21 is reflected in its expression levels in different cancer types compared with normal tissue (Figure 2a). Another interesting issue is the variability in the expression levels of CDKN1A/p21 among patients, for example, in the TCGA breast invasive carcinoma dataset (Figure 2c). This difference among patients could be used as a predictive tool for both outcomes and disease prognosis. Nevertheless, its binary role in cell functions, such as proliferation, apoptosis, stem cell regulation, and DNA damage response, make it clear that a simple intervention targeting CDKN1A/p21 in general may not be successful.

It is well established that CDKN1A/p21 interacts with the replication machinery by binding to CDKs and stopping the cell cycle. Apart from this, it is also an important regulatory factor in the control of gene expression via its interaction with transcriptional factors, such as C-Myc, the DREAM complex, the RB–E2F complex [79,80,81], and p53 [82]. There is strong evidence that CDKN1A/p21 is also a transcriptional factor itself [83,84], binding directly to chromatin and regulating gene expression. Another major function of CDKN1A/p21 is the regulation of DNA damage response (DDR), mainly by binding to PCNA and preventing interaction with DDR components [73]. Despite its inhibitory role, CDKN1A/p21 levels initially increase, in order for the cell cycle arrest and DDR to begin [77]. After the initial increase, the protein is either actively degraded [88], resulting in the initiation of the DNA repair process, or the cell becomes apoptotic or senescent. Despite the fact that cytoplasmic CDKN1A/p21 is mostly known to inhibit apoptosis by binding to pro-caspase 3 and ASK1 [151,152], nuclear CDKN1A/p21 induces apoptosis via complexing with p53 [156]. Nuclear CDKN1A/p21 also binds to CDKs, deactivating their pro-apoptotic function [153,154,155]. Therefore, the decision between apoptosis and survival is dependent on the levels of CDKN1A/p21, the specific timing of the cell cycle, and its cellular localisation. Interestingly, the levels of CDKN1A/p21 after DNA damage do not always increase as discussed previously regarding Translesion DNA Synthesis (TLS) [78,79]. TLS is a well-conserved process by which the cell can bypass DNA damage and continue the cell cycle, mediated by specialised polymerases; this process can result in tumour resistance to DNA-damaging reagents during cancer treatment [174,175,176].

It becomes clear that the regulation of the CDKN1A/p21 levels and its localisation in the cell have to be precisely regulated to prevent genomic instability (Figure 4). CDKN1A/p21 plays a regulatory role in almost all parts of the DDR and consequently determines the cell fate following damage. Also, CDKN1A/p21’s role in TLS and apoptosis suggests that it may be involved in the mechanism of drug resistance to chemotherapeutic reagents. Considering that a change in the levels and cellular localisation of CDKN1A/p21 can shift the balance in favour of cell survival against apoptosis, measurement of CDKN1A/p21 could be considered as a possible predictive marker for identifying patients with an increased risk of resistant disease. 

Acquired resistance to chemotherapy is not considered a characteristic of all cancer cells, and presents more commonly within the cancer stem cell (CSC) niche. Drug resistance depends on the cell’s ability to tolerate stress and continue the cell cycle. Thus, it is not surprising that the function of CDKN1A/p21 is important for the conservation of stem cell properties in tumours [135]. In general, the loss of CDKN1A/p21 leads to the upregulation of stem cell properties [114,137,138]. A combined treatment of DNA damaging drugs and reagents aimed at the upregulation of CDKN1A/p21 may be beneficial for patients, especially in cancers where recurrence is frequent, such as breast cancer. Another interesting role of CDKN1A/p21 in cancer is regarding EMT. There is evidence that CDKN1A/p21 negatively regulates EMT [113,114], most probably by interacting with EMT transcriptional factors such as ZEB1 [119] and Snail1 [120]. The exact mechanisms via which it interacts with these and other transcriptional factors still needs to be better understood. 

CDKN1A/p21 was originally described as an inhibitor of cell cycle binding to CDKs. Over the years, a lot of other functions, from DNA repair to apoptosis and senescence, were discovered to be part of its repertoire. It would be more suitable to say that CDKN1A/p21 is a regulator of cell fate. This regulation takes place both at the protein and gene expression levels, with the latter still not being well understood. CDKN1A/p21 plays a role in multiple processes important for cancer progression (cell cycle control, DNA damage, apoptosis, senescence, stem cell fate, EMT); however, its action can be both pro- and anti-tumorigenic. It varies depending on the cellular localisation, temporal regulation of its expression, total protein levels, as well as the intrinsic characteristics and gene expression signatures of the specific tissue and cell type. Given its central position in the regulation of cell fate, it makes CDKN1A/p21 a very promising target for cancer treatment. Therefore, CDKN1A/p21 is definitely a part of the problem, but it could also be a part of the solution, especially for breast cancer, where acquired drug resistance and metastasis are responsible for the majority of deaths. However, as discussed, given the dual role of CDKN1A/p21, a complete inhibition or silencing is unlikely to be beneficial. Therefore, a clearer understanding of the specific mechanisms, pathways, and regulators of its function is required so that it may be possible to develop novel specific therapeutic strategies.

## Figures and Tables

**Table 2:** Described actions of CDKN1A/p21 in different cancer studies.

Cancer Type	Mechanism	Description	Ref
Colorectal	LMNB2 binding to the P3- and P6-binding regions of the CDKN1A promoter	Regulation of CDKN1A promoter by LMNB2 results in silencing of p21 expression and promotes cell proliferation in CRC tissues.	[42]
Lung	Suppression of CDKN1A via promoter methylation	Transcriptional inactivation of CDKN1A in lung cancer cell lines and MPM.	[37]
Prostate	Methylation within the CDKN1Apromoter, at the 5′ end of a CpG island and STAT1-binding site	Inactivation of CDKN1A expression by methylation promotes cell proliferation and DNA replication in metastatic prostate cancer.	[38]
Breast	Aberrant hypermethylation of CDKN1A promoter	Increased risk of breast cancer in hypermethylated CDKN1A promoter reduces CDKN1A mRNA expression in all age groups. Inhibition of G1 arrest.	[39]
Lymphoblastic leukaemia	Hypermethylation of CpG islands within the CDKN1A promoter regions led to a decrease in CDKN1A expression	Loss of CDKN1A is associated with disease progression and poorer DFS. Selective growth advantage and accelerated onset of other pathways.	[40]
Oligodendrogliomas	In the nucleus, p21 promotes ODG by stabilising cyclin D1–cdk4. Cytosolic p21 binds procaspase 3, desensitising apoptotic stimuli	PDGF signalling produces p21 accumulation in ODG, which associates with cyclinD-cdk4 complexes and increases proliferation. Binding to cytosolic components reduced apoptosis.	[46]
Renal	CPEB4 binds to the CDKN1A transcripts and stabilises its mRNA, increasing CDKN1A expression	Increasing CDKN1A expression modulates RCC cell proliferation by inhibiting cell cycle progression due to G1 cell cycle arrest.	[44]
Lymphomas	p21 provides survival to TNF- or Fas-triggered apoptosis by binding and inhibiting caspase-3	Absence of p21 increases lifespan due to reduced incidence of thymic lymphomas. p21-proficient lymphomas have faster growth due to lower apoptotic rate.	[47]
Hepatocellular	p21 is stabilised by the repression of ubiquitin-proteasome proteolysis due to CMTM6 binding	Extension of CDKN1A half-life by CMTM6 suppresses HCC cell proliferation, sensitising patients to treatment and increasing survival rates.	[45]
Endometrial	p21/CDKN1A gene transcription is repressed via SOX2 binding to the CDKN1A promoter	Inhibition of CDKN1A by SOX2 overexpression correlates with poor prognosis in endometrial cancer due to cell cycle progression.	[43]
Pancreatic	DNMT1 silencing results in the upregulation of p21 expression	Upregulation of p21 decreases cell proliferation in pancreatic cancer cell lines.	[41]

## Data Availability

All references associated with the public databases analysed in this review are listed in the reference section.

## Supplementary Materials

The following supporting information can be downloaded at https://www.mdpi.com/article/10.3390/ijms242417488/s1.

## Abbreviations

AKT	AKT serine/threonine kinase 1
AML	Acute Myeloid Leukemia
ASK1	Apoptosis signal-regulating kinase 1
BER	Base Excision Repair
BCSC	Breast cancer stem cell
CDK	Cyclin-dependent kinase
CDKN1A	Cyclin-dependent kinase inhibitory protein 1A
CHEK1	Serine/threonine-protein kinase Chk1
CIP	CDK-interacting protein
CMTM6	CKLF-Like MARVEL Transmembrane Domain-Containing Protein 6
CMS4	Consensus molecular subtype 4
CPEB4	Cytoplasmic polyadenylation element-binding protein 4
CRC	Colorectal cancer
CSC	Cancer stem cell
DAPK1	Death-associated protein kinase 1
DFS	Disease-free survival
DNMT1	DNA Methyltransferase 1
DDR	DNA damage repair
DREAM	Dimerisation partner, RB-like, E2F, and multi-vulval class B
DYRK1B	Dual-specificity tyrosine-phosphorylation-regulated kinase 1B
EMT	Epithelial–mesenchymal transition
GSK	Glycogen synthase kinase
HCC	Hepatocellular carcinoma
HDAC	Histone Deacetylases
H and N	Head and Neck
HR	Homologous Recombination
KIP	Kinase inhibitor protein
LATS2	Serine/threonine-protein kinase LATS2
LEC	Lymphatic endothelial cells
LKB1	Serine/threonine-protein kinase STK11
LMNB2	Lamin B2
MAPK	MAP kinase-activated protein kinase
MELK	Maternal embryonic leucine zipper kinase
MPM	Malignant pleural mesotheliomas
MMR	Mismatch repair pathway
NER	Nucleotide excision repair
NHEJ	Non-homologous end joining
NSCLC	Non-small-cell lung carcinoma
NUAK1	NUAK family SNF1-like kinase 1
NUDT5	Nudix hydrolase 5
ODG	Oligodendrogliomas
PARP1	Poly-ADP-ribose polymerase
PCNA	Proliferating cell nuclear antigen
PDGF	Platelet-Derived Growth Factor
PIM1	Serine/threonine-protein kinase pim1
PKA/B	Protein kinase B (Akt)
PRKCD	Protein kinase C delta type
PRMT1	Protein arginine N-methyltransferase 1
PVT1	Plasmacytoma Variant Translocation 1
RB	Retinoblastoma
RCC	Renal cell carcinoma
SOX2	Sex-determining region Y-box 2
STK38	Serine/threonine-protein kinase 38
STAT1	Signal transducer and activator of transcription 1
TLS	Translesion DNA synthesis
TSA	Trichostatin A
ZEB1	Zinc finger E-box-binding homeobox 1
